# Nonsense Mutations in Rare and Ultra‐Rare Human Disorders: An Overview

**DOI:** 10.1002/iub.70031

**Published:** 2025-06-06

**Authors:** Emanuele Vitale, Davide Ricci, Federica Corrao, Ignazio Fiduccia, Ilenia Cruciata, Pietro Salvatore Carollo, Alessio Branchini, Laura Lentini, Ivana Pibiri

**Affiliations:** ^1^ Department of Biological, Chemical and Pharmaceutical Sciences and Technologies University of Palermo Palermo Italy; ^2^ Department of Life Sciences and Biotechnology and LTTA Centre University of Ferrara Ferrara Italy

**Keywords:** genetic rare diseases, nonsense mutations, premature termination codons, translational readthrough

## Abstract

Over 7000 rare diseases have been described, collectively affecting 350 million people worldwide. Most of these conditions result from nonsense mutations, representing approximately 10% of all genetic mutations associated with human inherited diseases. Nonsense mutations convert a sense codon into a premature termination codon (PTC), leading to premature translation termination and the production of truncated, nonfunctional proteins. This results in a loss‐of‐function phenotype in many genetic disorders, contributing to the disease's severity and progression. The molecular mechanisms of PTC formation involve various genetic alterations, including single‐nucleotide changes, frameshifts, and splicing mutations. The nonsense‐mediated mRNA decay (NMD) pathway degrades mRNAs containing premature termination codons (PTCs). In contrast, 25% of PTC mRNAs, depending on the PTC position and cellular context, can evade NMD, resulting in the synthesis of truncated proteins. A termination codon during translation is essential for proper protein synthesis, and translational readthrough—a process in which the ribosome bypasses the PTC and reaches the natural stop codon—may restore some level of protein function. The effectiveness of readthrough depends on the surrounding genetic context and the type of amino acid incorporated at the PTC position. This review aims to explore the molecular characteristics of nonsense‐related diseases (NRDs), including cystic fibrosis, hemophilia, Fabry disease, choroideremia, Usher syndrome, Shwachman‐Diamond syndrome, and certain hereditary neuropathies and cancers.

**Abbreviations**
CF: cystic fibrosis
CHM: choroideremia
HA/B: haemophilia A/B
LSDs: lysosomal storage disorders
NMD: nonsense‐mediated decay
NRDs: nonsense‐related diseases
PTC: premature termination codon
SDS: Shwachman‐Diamond syndrome
USH: Usher syndrome

## 1 Introduction

Over 7000 rare genetic diseases have been identified, collectively affecting an estimated 350 million people worldwide [1]. Approximately 10% of all the genetic mutations associated with human‐inherited diseases are nonsense mutations [2].

Nonsense or stop mutations are single‐base pair substitutions that affect gene coding regions, converting an mRNA sense codon into an in‐frame premature termination codon (PTC) [3]. The molecular mechanisms underlying the formation of a PTC are varied, including single‐nucleotide changes, frameshifts, or splicing mutations, which result in alterations to the three‐letter code of the open reading frame (ORF). Typically, an ORF contains an AUG codon as the initiation signal for the translation process and one of the three termination codons, UGA, UAA, or UAG (opal, ochre, and amber, respectively), which delimit the region to be translated. Unfortunately, a PTC resulting from a nonsense mutation causes premature protein translation termination and the production of a truncated non‐functional polypeptide, exacerbating the loss of function [4].

Two intracellular quality control mechanisms typically recognize this event. On the one hand, nonsense‐mediated mRNA decay (NMD) selectively degrades mRNAs that harbor premature translation termination codons (PTCs), ensuring the quality of translated transcripts [5]. NMD occurs both in the nucleus and in the cytoplasm and is activated by the presence of a PTC that is not located in the last exon and is positioned at least 50 nt upstream of the penultimate exon‐exon junction. The NMD mechanism involves the UPF1, UPF2, UPF3, SMG‐1, SMG‐5, SMG‐6, and SMG‐7 factors, which lead to mRNA degradation through decapping complex‐mediated degradation and 3′–5′ decay [6].

This process plays a crucial role in preventing the production of aberrant polypeptides while simultaneously decreasing mRNA levels, thereby reducing the availability of PTC‐containing transcripts that could otherwise be used to restore expression through the molecular mechanism known as translational readthrough [7].

On the other hand, up to approximately 25% of PTC‐containing mRNAs can evade the NMD pathway, resulting in the production of truncated proteins that are detected by the endoplasmic reticulum (ER) quality control system, leading to their removal, often in a proteasome‐dependent manner [5]. The complexity of a nonsense mutation's effect also depends on the genetic context surrounding the PTC. Indeed, the type of PTC insertion needs to be considered, which accounts for 36.1% of UGA, 41.9% of UAG, and 22% of UAA codons [3].

However, the presence of a termination codon is necessary during usual mRNA translation because its entry in the A‐site of the ribosome attracts translation termination factors, such as eRF1 (eukaryotic release factor 1), allowing the detachment of the newly synthesized polypeptide, its post‐translational modifications, and its proper cellular localization [8].

The mRNAs pool evading NMD can be translated by bypassing the PTC and reaching the proximal natural stop codon, thanks to ribosome misreading activity, in a process known as translational readthrough [9, 10]. Additionally, in this case, the nucleotides surrounding the PTC and the genetic context, such as the position of the stop codon, appear to influence readthrough levels and play a crucial role in amino acid incorporation [11]. For example, if there is a cytosine at the +4 position (the nucleotide immediately following the stop codon), readthrough levels are increased [12]. Translational readthrough is also influenced by the type of amino acid incorporated at the PTC position, which affects the final protein product [3, 13].

**FIGURE 1:**
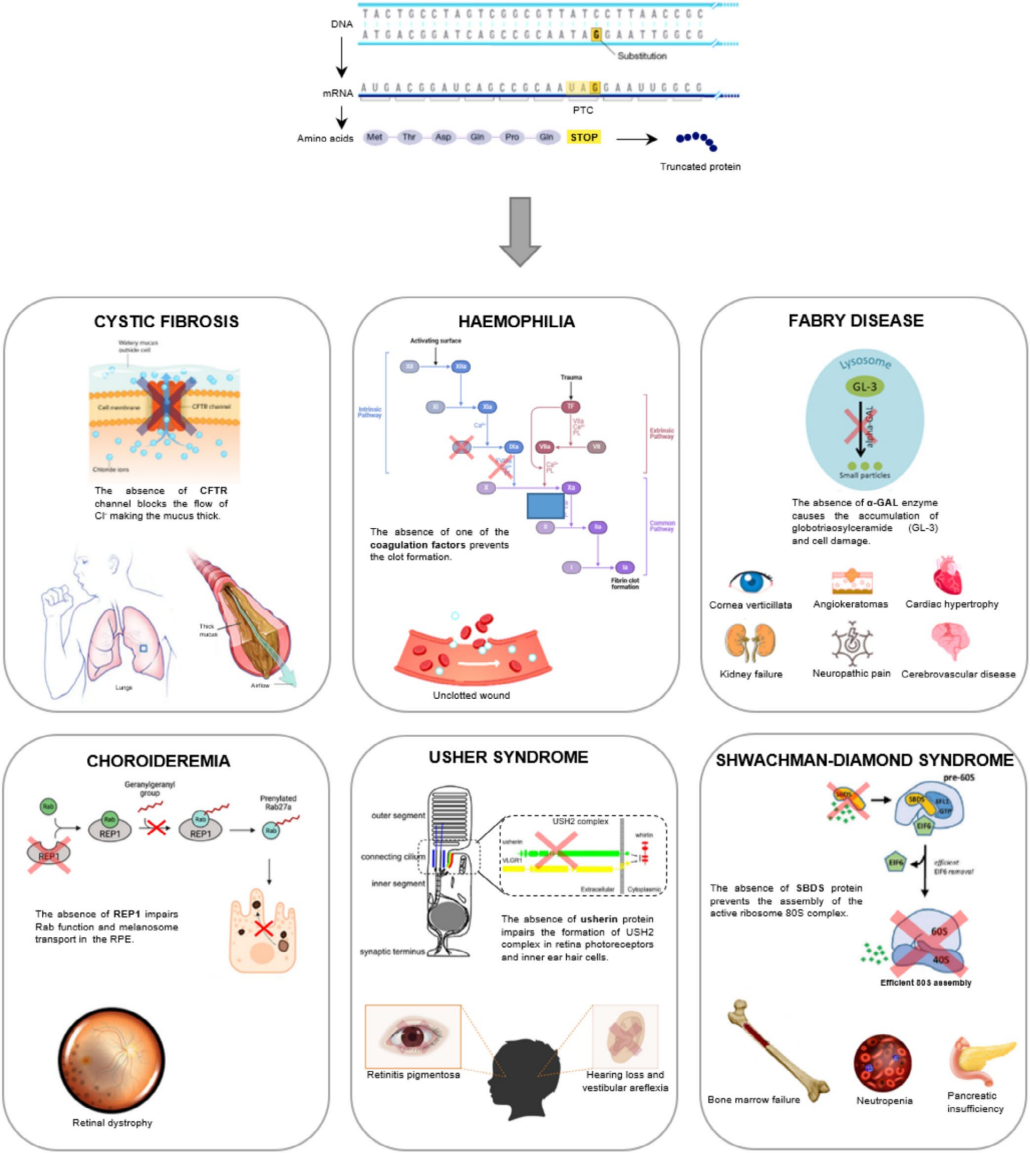
Some of the nonsense‐related diseases (NRDs) described in the present review. At the top is a schematic representation of a general nonsense mutation that causes the formation of a PTC in the mRNA sequence, resulting in the production of a truncated protein. In each box, the protein involved in the described NRD and the phenotypic consequence of its absence are described.

The lack of protein expression resulting from a nonsense mutation is the primary cause of a severe phenotype in most nonsense‐related diseases (NRDs). Among NRDs, cystic fibrosis, hemophilia A and B, Fabry disease, choroideremia, Usher syndrome, Schwachman‐Diamond syndrome, certain hereditary neuropathies, and some cancers with p53 involvement can be mentioned (Figure 1).

The scope of this review is to reassess the molecular characteristics and mechanisms of the disorders cited above, with a focus on nonsense mutations that affect the genes involved in each disease.

## 2 Rare and Ultra‐Rare Nonsense‐Related Diseases: Molecular Characteristics and Mechanisms

### 2.1 Cystic Fibrosis

Cystic fibrosis (CF) is an autosomal recessive genetic disease that affects approximately 160,000 people worldwide [14]. Among these individuals, 10% of CF patients harbor nonsense mutations [15]. The molecular mechanism was described in 1989 by Riordan et al., who identified the Cystic Fibrosis Transmembrane Conductance Regulator (CFTR) gene as the cause of the onset of the pathology [16]. The *CFTR* gene (OMIM #602421) is located on the long arm of chromosome 7 (position 7q31.2), contains 27 exons, and encodes a 170 kDa glycoprotein of 1480 amino acids [17]. The CFTR protein is a member of a large family of membrane transport proteins, the ATP‐binding cassette (ABC) transporters, and is responsible for transporting chloride (Cl^−^) and bicarbonate (HCO_3_^−^) [18]. Its structure can be divided into three principal parts: two transmembrane domains (TMDs), each composed of six alpha helices; two intracellular nucleotide‐binding domains (NBDs), responsible for ATP binding and hydrolysis, which are necessary for channel activation; and a regulator domain (R), located between the two NBDs, whose phosphorylation by protein kinase A (PKA) or C (PKC) is involved in channel gating [19]. The CFTR protein is localized in the apical membrane of epithelial cells in several organs, including the lungs, liver, pancreas, sweat glands, intestines, and vas deferens, which explains its involvement in various physiologic dysfunctions [18].

Approximately 2100 mutations in the CFTR gene have been identified and categorized into six classes based on the severity of the phenotype [20]. Nonsense mutations, in particular, are classified in class I, which can be divided into two subclasses. Class IA includes large deletions, frameshifts, or specific splicing mutations (e.g., Dele2,3, 1717 + 1G>A), which are characterized by the absence of CFTR mRNA and are still unrecoverable from a therapeutic perspective. Class IB includes nonsense mutations, whose presence causes the lack of CFTR protein due to mRNA suppression by the NMD pathway or to the degradation of the truncated protein by the proteasome system [21]. Some examples of the most common nonsense mutations in CF are p.G542X (2%–4% of the alleles), p.W1282X, p.R1162X, and p.R553X [20]. Nowadays, several studies focus on targeting nonsense mutations in cystic fibrosis (CF). As reported by Ricci et al., different therapeutic approaches are being explored to address this disease, including post‐translational strategies, gene editing, and mRNA‐based therapies [22]. The first category includes anticodon‐engineered tRNA (ACE‐tRNA), which has already demonstrated the ability to rescue PTC‐containing mRNA from nonsense‐mediated decay (NMD), and more importantly, restore functional protein expression in 16HBEge cells harboring three common CF mutations: G542X, R1162X, and W1282X [23]. Another approach in this category was the use of translational readthrough‐inducing drugs (TRIDs), such as PTC124 (ataluren), which initially showed promising results in suppressing nonsense mutations but failed to meet primary efficacy endpoints in Phase III clinical trials for CF [24]. Additionally, 2,6‐diaminopurine (DAP) has shown high efficiency in promoting readthrough of the UGA stop codon, with promising results demonstrated in both mouse models and patient‐derived organoids [25]. ELX‐02, a synthetic aminoglycoside, also belongs to this group and in 2023 successfully completed a Phase II clinical trial for CF, reporting significant improvements in patient conditions following treatment [26]. The second category involves the use of CRISPR/Cas9 gene editing against CF nonsense mutations. This technology has been employed to correct homozygous mutations such as R785X and W1282X, restoring CFTR functionality in patient‐derived organoids, which subsequently showed increased forskolin‐induced swelling [27]. The third category includes systems designed to introduce wild‐type CFTR mRNA sequences directly into pulmonary cells via nebulization. For instance, SORT lipid nanoparticles (LNPs) have been developed and have shown promising in vitro results for CF treatment [28]. Finally, VX‐552 an mRNA aerosol therapy has been developed and is currently undergoing a Phase I clinical trial to evaluate its safety in CF patients (NCT05668741).

### 2.2 Haemophilia and Rare Coagulation Disorders

Coagulation factors are involved in the coagulation cascade, a finely tuned process that ultimately leads to clot formation [29]. Defects in one of these components are associated with coagulation disorders characterized by different severities depending on the specific factor involved and its residual levels [30].

In this scenario, nonsense mutations, relatively frequent (10%–14%) in X‐linked deficiencies of factors (F)VIII (haemophilia A, HA; OMIM #306700) and (F)IX (haemophilia B, HB; OMIM #306900) [31, 32, 33, 34], are associated with severe forms in all bleeding disorders. Unexpectedly, despite their general association with severe phenotypes, some exceptions have been observed for PTCs affecting the carboxyl‐terminal region of FVII and FX, where missense/nonsense mutations in homologous coagulation factors are predicted to be detrimental [35, 36, 37, 38, 39, 40, 41].

Notably, the low therapeutic threshold required to ameliorate the clinical phenotype in coagulation factor disorders (in the range of 2%–5% of normal protein levels) is compatible with the predicted extent of rescue expected from correction approaches such as readthrough or even others resulting in minimal expression levels [42, 43, 44].

This drove the first attempts for therapeutic approaches based on readthrough in patients with PTCs in *F8* and *F9* [45] as well as *F7* (FVII deficiency; OMIM #227500) [46, 47] genes or in HB mouse models [48], which showed sub‐therapeutic levels or moderate phenotype amelioration, suggesting minimal drug (gentamicin) effects, low suppression efficiency (nucleotide context) and/or amino acid substitutions incompatible with function (protein context). In line with this, a phase 2a clinical trial on readthrough induction with orally administered Ataluren [49] in HA and HB patients (NCT00947193) started in 2009 but was suspended in 2011.

Studies in experimental models of coagulation disorders have helped dissect the interplay between sequence and protein contexts, including the influence of PTC localization within the coding sequence. These studies provide insights into the impact of the inserted amino acids at the PTC site upon readthrough. In rare coagulation disorders, interesting findings have been obtained in complementary cellular models of FV deficiency [50]. Interestingly, the integration of results from expression studies involving nonsense and predicted readthrough‐derived missense variants [13, 51] indicated a significant functional rescue related to (i) favorable nucleotide contexts, (ii) degree of re‐insertion of the original residue, (iii) rare gain‐of‐function effects resulting from missense variants, and (iv) favorable localization of PTCs in regions (i.e., signal peptides or the large FVIII B domain) whose removal during protein processing promotes the production of full‐length proteins with wild‐type features. Additionally, the findings highlight negligible readthrough effects for PTCs with low suppression efficiency and/or those affecting positions where amino acid substitutions are not tolerated [52, 53, 54, 55, 56, 57].

These elements provide a background that contributes to a retrospective interpretation of studies in patients. In particular, gentamicin administration in haemophilia patients with *F8* (p. R446X, p. S1414X, p. R2135X) and *F9* (p. R252X and p. R379X) PTCs resulted in a transient improvement of coagulant activity in those bearing the p. S1414X (HA) and p. R379X (HB) mutations. Interestingly, despite the low suppression efficiency of the *F8* p. S1414X PTC (UAA) in predicting the production of missense variants, the observed rescue, albeit moderate, is attributed to its localization within the large FVIII B domain, which tolerates amino acid substitutions [58, 59].

### 2.3 Lysosomal Storage Disorders

Lysosomal storage disorders (LSDs) comprise a panel of rare inherited metabolic diseases with different prevalences, associated with deficiencies in key enzymes, and characterized by the accumulation of undegraded substrates within lysosomes in multiple tissues and organs [60, 61]. Nonsense mutations may represent a relevant target for correction strategies in LSDs based on readthrough induction, given the relatively low threshold required to alleviate the associated disease phenotype, potentially [62]. Several examples, involving different experimental models, have been reported for LSDs [63, 64, 65, 66, 67, 68, 69, 70, 71, 72, 73], including Mucopolysaccharidosis I‐Hurler (MPS I‐H) and Fabry disease (FD), where nonsense mutations are relatively frequent.

MPS I‐H (OMIM #607014) is a severe form of α‐L‐iduronidase (IDUA) deficiency, characterized by the accumulation of glycosaminoglycans [74]. The Q70X and W402X are the two most common nonsense mutations in Caucasians [75]. Readthrough induction revealed a relevant rescue for the Q70X (CAG>UAG) but partial for the W402X (UGG>UAG) variant in different expression models [69, 76, 77], an observation that can be interpreted as a contribution of different susceptibility of PTC nucleotide contexts (UAGC>UAGG) [78] and the predicted (Q79X) or unpredicted (W402X) insertion of the authentic residue in the subset of amino acids introduced by readthrough over UAG PTCs [13, 51]. These first findings prompted attempts, albeit with variable efficiency outputs, in a knock‐in mouse model bearing the W392X PTC [79, 80], corresponding to the human W402X PTC, as well as by challenging NMD attenuators to increase mRNA transcripts as potential readthrough substrates [81].

FD (OMIM #301500) is a rare X‐linked LSD caused by a deficiency of the homodimeric α‐galactosidase A (α‐gal A) enzyme [82], encoded by the *GLA* gene, and failures in the metabolism of glycosphingolipids [83]. Depending on residual enzymatic activity, FD patients may exhibit either a severe/classic phenotype (characterized by less than 1% activity or complete loss of function) or a late‐onset phenotype (with residual activity) [84]. In the *GLA* mutational pattern, relatively frequent nonsense mutations exhibit the highest association with the classic form of familial dysautonomia (FD). Interestingly, due to the oligomeric nature of α‐gal A, FD provides a paradigmatic model for oligomeric proteins, where the amino acid substitutions introduced by readthrough may exert unpredictable effects on biosynthesis and function by impacting the enzyme's quaternary structure.

A first attempt to correct FD nonsense mutations by readthrough induction was provided in patients' fibroblasts bearing the frequent R227X (UGA) variant [69]. Despite the potentially high suppression efficiency of the PTC, the involvement of the R2227 residue in the formation of the α‐gal A active site makes amino acid substitutions at this position unlikely to be compatible with rescuing enzyme function, contributing to the explanation of the low responsiveness observed.

### 2.4 Choroideremia

Choroideremia is an X‐linked recessive disease caused by mutations in the CHM gene, a member of a large class of retinal disorders. This retinal dystrophy causes the progressive degeneration of choriocapillaris, photoreceptors, and retinal pigment epithelium (RPE). Notably, its incidence swings between 1:50,000 and 1:100,000 [85].

Nowadays, more than 500 mutations are known to affect the *CHM* gene, including nonsense (34%), deletions (2%), missense (8%), frameshift (51%), insertions (1%), indels (1%), other (1%) and unknown (2%) [86]. Currently, 116 nonsense mutations have been recorded in *ClinVar* for the *CHM* gene, and all are pathological. The most diffuse are p.R239X, p.R253X, p.R267X, p.R270X, and p.R293X [87]. The *CHM* gene (OMIM #300390) is located on the long arm of the X chromosome at position Xq21.2. It encodes Rab escort protein 1 (REP1), a 95 kDa ubiquitous protein of 653 amino acids. REP1 protein is constituted by two main domains: the first one is cylindrical and consists of six α‐helices and four β‐sheets; the second one, called “domain II” is smaller and consists of five α‐helices [88]. REP1 is an escort protein involved in the intracellular trafficking and prenylation of polypeptides, a post‐translational modification essential for the correct functionality of Rab proteins. Additionally, REP1 binds the newly synthesized Rab proteins, presenting them to Rab geranyl‐geranyl‐transferase II (RabGGTII) for prenylation. Structural studies have demonstrated a close association between the REP1 domain II and the α subunit of RabGGTII [89]. In particular, in the retina, the absence of REP1 results in the lack of functional Rab protein, which is strictly linked to melanosome transport in the RPE and the transfer of different proteins into photoreceptors, leading to atrophy and cell death in these specific regions [90]. Despite the widespread presence of REP1, the disorder primarily affects the eyes. Indeed, the presence of a REP2 protein, encoded by a CHM‐like gene, addresses the absence of REP1 in all other tissues. Currently, there are no approved treatments for nonsense mutations in choroideremia. However, several research groups are increasingly focusing their efforts on this disease. Various preclinical studies are investigating strategies that target nonsense mutations. Over the past decades, PTC124 and its analogue PTC414 have shown promising results as readthrough agents capable of restoring REP1 expressions in a zebrafish model of choroideremia. In contrast, in p. K258* fibroblasts and iPSC‐derived RPE cells with UAA PTC, treatment with PTC124 did not restore REP1 protein expression or function [86]. Among the different therapeutic approaches, adeno‐associated virus (AAV)‐mediated gene therapy is gaining increasing attention. Several AAV‐based gene therapies are currently in clinical development [86, 91], with some having progressed to phase III clinical trials. These have demonstrated average visual improvements, although important challenges remain, such as optimizing the therapeutic window, enhancing cell‐specific targeting, and minimizing viral toxicity [92]. Lastly, data from ongoing and completed clinical trials provide critical insights that will inform the design of future studies, including optimal treatment timing, patient selection, vector engineering, and the choice of outcome measures [93].

### 2.5 Usher Syndrome

Among NRDs, Usher syndrome (USH) is the most common form of hereditary deaf‐blindness, affecting individuals at a rate of 4:100,000 to 17:100,000 [94]. USH is considered a ciliopathy with an autosomal recessive hereditary nature. It is characterized by sensorineural hearing loss (SNHL), retinitis pigmentosa (RP), and vestibular areflexia (VA) [94]. USH is genetically and clinically heterogeneous, and its classification is based on the severity of symptoms, the onset and progression of symptoms, and the involved genes, resulting in three distinct categories: USH1, USH2, and USH3.

USH1 is the most severe form, accounting for 25%–44% of all USH patients [94]. It is characterized by severe congenital SNHL, VA, and RP with a prepubertal debut. USH2 is the most common form, accounting for more than 50% of all USH cases, and is characterized by less severe symptoms compared to USH1 [95]. In USH2 patients, the sensorineural hearing loss (SNHL) is moderate at low frequencies and severe at higher frequencies. The symptoms related to cone‐rod dystrophy typically start later, around the second decade of life. Vestibular function is generally normal. USH3 is the least common form, accounting for only 2%–3% of USH cases, although it is relatively common among certain populations, such as the Finnish and Ashkenazi Jewish [95]. In USH3 patients, a benign sensorineural hearing loss (SNHL) typically develops at higher frequencies after the age of 35, retinitis pigmentosa (RP) arises after the second decade of life, and vestibular dysfunctions affect only approximately half of the patients [94]. Ten genes have been associated with USH: *MY7OA*, *PCDH15*, *CDH23*, *USH1G*, *CIB2*, and *EPSIN* give rise to USH1; *USH2A*, *ADGRV1*, and *WHRN* give rise to USH2; and *CLRN1* gives rise to USH3 [96]. All of them encode proteins widely expressed in sensory cells, such as outer hair cells (OHC), inner hair cells (IHC), and vestibular hair cells (VHC) in the inner ear, as well as photoreceptive cells (cone and rod) in the retina. Numerous nonsense mutations affect USH‐associated genes. In USH1, several nonsense mutations have been identified and annotated in public mutation databases in genes associated with the disorder as USH1B (*MYO7A*; p. R694X, p. R2106X, p. G369X, p. R805X). These mutations contribute to hearing loss, balance issues, and progressive vision loss. The primary nonsense mutations in the USH2A gene, associated with Usher syndrome type 2 (USH2), include variants that introduce premature stop codons, leading to a truncated, nonfunctional protein. Some of the most common mutations reported in *ClinVar* and other databases include p.E767X (one of the most frequent mutations in the USH2A gene), p.W3955X, p.R3043X, p.G34X, and p.T2853X. These mutations are classified as pathogenic, resulting in loss of function of the usherin protein, which is essential for auditory and retinal function. Several preclinical studies are currently underway to investigate nonsense mutations in USH. Lastly, NB30, NB54, and PTC124 readthrough activity were evaluated to restore full‐length harmonin protein from USH1C transcripts carrying the p. R31X nonsense mutation. All compounds promoted read‐through in vitro, with NB54 and PTC124 demonstrating the highest efficiency and restoration of protein function. Additionally, in murine and human retinal explants, both NB54 and PTC124 exhibited excellent biocompatibility and effective harmonin expression, whereas NB30 showed increased toxicity. In vivo studies confirmed that NB54 and PTC124 induced comparable levels of full‐length harmonin. These findings support the therapeutic potential of NB54 and PTC124 for nonsense mutation‐associated retinal disorders [97, 98].

### 2.6 Shwachman‐Diamond Syndrome

Shwachman‐Diamond syndrome (SDS) is a rare autosomal recessive genetic disease, first described in 1964 by Dr Shwachman and Dr Diamond, and classified as a ribosomopathy [99]. SDS is a multi‐organ disease that affects about 1:100,000 to 1:150,000 newborns every year (data from the Italian SDS registry), even though this frequency could be underestimated due to difficulties in the diagnosis within the first year of life and incomplete knowledge to recognize the genetic defects [100]. The main affected organ is bone marrow, which, in most cases, is hypocellular and could give rise to marrow bone failure syndrome, the primary cause of death in SDS patients. In addition, they are predisposed to develop myelodysplastic syndrome (MDS) with a high risk of progression to acute myeloid leukemia (AML) [101]. Severe neutropenia is also present in almost all SDS patients [102, 103], and other recently identified symptoms are cognitive impairment, liver dysfunction, and cardiac abnormalities that are mild and/or resolved in young age [104, 105]. Another hallmark of SDS is pancreatic insufficiency, which leads to the malabsorption of essential nutrients, including vitamin D and K [100].

In 90% of cases, SDS patients carry mutations in the *SBDS* gene (OMIM #607444), located on chromosome 7 at position 7q11 and encoding for the SBDS ribosome maturation factor. Frequent mutations for this gene are c.258 + 2T>C and a dinucleotide alteration 183‐184TA>CT that introduces a PTC causing the p. K62X nonsense mutation [102]. The most common condition in SDS patients is heterozygosity, where one allele presents the c.258 + 2T>C mutation, and the other presents the 183‐184TA>CT mutation. Another pathological condition is the homozygosity for the c.258 + 2T>C mutation. Precisely, homozygous patients for the c.258 + 2T>C mutation have been observed, as well as heterozygotes for the c.258 + 2T>C mutation and other less frequent mutations. Conversely, no homozygous individuals for the 183‐184TA>CT mutation have been observed, indicating that the total loss of function of the SBDS protein may be fatal to life, as confirmed by animal models [100, 106]. The SBDS protein owns three principal domains: domain I (S2‐S96), also called FYSH (Fungal Yhr087wp Shwachman) domain; domain II (D97‐A170), a three‐helical central domain; domain III (H171‐E250), the C‐terminal domain with a ferredoxin‐like fold [107]. SBDS protein stimulates the GTP hydrolysis by the GTPase elongation factor‐like 1 (EFL1): this produces a conformational change in EFL1 that leads SBDS protein to rotate and causes a destabilization of the linkage between the eukaryotic initiation factor 6 (eIF6) and 60S ribosomal subunit [102]. This destabilization is fundamental for the release of eIF6 and for the binding of the 40S subunit to the 60S subunit to generate the active ribosome complexes 80S. So, when the SBDS retentions of eIF6 are not performed, this provokes the impairment of the translational machinery, leading to the activation of p53 [102].

Moreover, other mutated genes can give an SDS‐like phenotype, such as *DNAJC21* (encoding for DnaJ heat shock proteins family member C21), *EFL1* (encoding for elongation factor‐like 1), and *SRP54* (encoding for signal recognition particle 54) [108], all proteins involved in the biogenesis and maturation of ribosomes, further strengthening the classification of SDS as ribosomopathy [102].

No specific treatment for SDS has been developed; therefore, patient management is based on treating clinical manifestations through a multidisciplinary approach [109]. Nevertheless, a series of preclinical studies are improving knowledge against nonsense mutation in SDS; in particular, PTC124 was evaluated in vitro and ex vivo in SDS cellular models carrying the c. 183‐184TA>CT nonsense mutation in the SBDS gene. Treatment with this TRID restored the full‐length SBDS protein, thereby improving 80S ribosome assembly and global protein synthesis, and significantly reducing the elevated p53 levels in hematopoietic cells. Furthermore, SBDS synthesis was restored in primary osteoblasts, suggesting broader tissue effects [110]. In addition, new derivatives of PTC124, namely NV848, NV914, and NV930 showed promising results, especially NV848 that effectively restored full‐length SBDS protein expression in lymphoblastoid cell lines, peripheral blood mononuclear cells (PBMCs), and periodontal ligament stem cells (PDLSCs) derived from SDS patients, and demonstrated in vivo excellent safety in zebrafish models, with no developmental toxicity or increase in apoptosis observed at concentrations up to 1 mM [99].

### 2.7 Hereditary Neuropathies

The peripheral nervous system can be affected by a group of inherited disorders, leading to hereditary neuropathies that are divided into four subcategories: (i) hereditary motor and sensory neuropathy, (ii) hereditary sensory neuropathy, (iii) hereditary motor neuropathy, and (iv) hereditary sensory and autonomic neuropathy.

Charcot–Marie–Tooth (CMT) disease represents a powerful example of common hereditary motor and sensory neuropathy because it is estimated to affect 1 in 2500 people [111]. CMT encompasses a diverse group of inherited neuropathies, predominantly characterized by autosomal dominant inheritance, in which peripheral motor and sensory neurons degenerate due to damage to myelinating Schwann cells (CMT type 1, CMT1) or to axons (CMT type 2, CMT2). Autosomal recessive inheritance or X‐linked forms of the CMT diseases have been diagnosed as well. Patients exhibiting late‐onset forms of CMT2 often carry a mutation in the membrane metalloendopeptidase (MME) gene, which can lead to dominant or recessive axonal CMT forms. The MME gene (OMIM #120520), located on chromosome 3q25.2, spans a region of approximately 99,536 bases and comprises 24 exons. The MME protein contains 750 amino acids and is a type‐II membrane‐anchored enzyme known to inactivate oligopeptides. Through whole‐exome sequencing, a novel nonsense mutation, Q522X, has been identified in the MME gene, inherited in an autosomal recessive manner. This mutation results in the loss of the C‐terminal region of the endopeptidase neprilysin (NEP) protein (OMIM #120520), which inactivates a broad spectrum of peptide substrates, such as enkephalins, neurokinin A, substance P, bradykinin, endothelin, somatostatin, and more others, including amyloid‐β (Aβ) protein, which accumulates in Alzheimer's disease (AD). Since the Q522X mutation in *MME* is a loss‐of‐function mutation, causing either protein truncation or mRNA degradation by NMD, it could be speculated that CMT patients are susceptible to AD. However, it has been investigated that *MME* mutations on their own are not sufficient for the development of early‐onset AD [112].

Furthermore, mutations affecting the myelin protein zero (MPZ) gene deliver vast hereditary neuropathies [113]. *MPZ* gene (OMIM #159440), located on chromosome 1q23.3 and spanning 7000 bases and 6 exons, encodes for the corresponding protein MPZ, a member of the immunoglobulin (Ig) superfamily, made of 219 amino acids, with an extracellular N‐terminal domain, a single transmembrane region, and a smaller positively charged intracellular region. It is primarily expressed in Schwann cells and plays a crucial role in myelination as an adhesion molecule, maintaining the continuity of contiguous wraps of myelin membrane through MPZ‐mediated homotypic interactions. P0Q215X, a nonsense mutation on the *MPZ* gene, causes severe congenital hypomyelination (CH). This mutation can evade the surveillance of the NMD mechanism, resulting in a shortened protein and altered trafficking to the non‐myelin plasma membrane, which in turn induces defects in the radial sorting of axons by Schwann cells. An experiment conducted on mouse models showed that P0Q215X acts through dose‐dependent gain‐of‐function [114].

### 2.8 Li‐Fraumeni Syndrome

Li‐Fraumeni syndrome (LFS) is an inherited genetic disorder characterized by a high predisposition to develop various malignant tumors at an early age. It is primarily caused by mutations in the *TP53* gene, which encodes the p53 protein—a crucial tumor suppressor. Autosomal dominant inheritance is caused by a single mutated copy of TP53, which is sufficient to increase the risk of cancer. High incidence of early‐onset tumors, including sarcomas (osteosarcoma, rhabdomyosarcoma), brain tumors (glioblastomas, medulloblastomas), early‐onset breast cancer, leukemia, and adrenal tumors (adrenocortical carcinoma). The *TP53* gene (OMIM #191170), located on the short arm of chromosome 17, encodes the transcription factor p53 (53 kDa, 393 amino acids), which is involved in suppressing tumor initiation [115]. Generally, p53 activity is kept low by the interaction with the MDM2 protein, which stimulates p53 proteasome degradation via ubiquitination. p53 activation occurs in response to various cellular stresses, including DNA damage and ribosomal stress. It involves the loss of MDM2 binding and post‐translational modifications, such as phosphorylation, on the p53 protein sequence, leading to subsequent cell cycle arrest, DNA damage repair, cell death, and changes in cell metabolism [116].

The *TP53* gene, also known as the “guardian of the genome”, is the most frequently mutated in various types of cancer [116]. Mutations in *TP53* negatively affect chemotherapy outcomes and patient prognosis [116]; therefore, therapeutic approaches to re‐establish wild‐type p53 activity are highly desirable. Most mutations in *TP53* are missense mutations (62.74%), but around 10% are nonsense mutations [117]. The most frequent *TP53* nonsense mutations found in cancers are p.R196X and p.R213X [118], resulting in a premature UGA stop codon in the mRNA sequence. p. R213X is, however, the mutation with the highest frequency found in various cancers, including lung squamous cell carcinoma, pediatric acute lymphoblastic leukemia, and primary breast carcinoma [119].

## 3 Emerging Therapeutic Approaches Against Nonsense Mutations

Several promising approaches for the treatment of nonsense mutations are currently under investigation or in clinical trials. These approaches can be categorized into three main strategies: gene editing, transfection, and post‐transcriptional gene recovery. Among the various gene editing methodologies under development, CRISPR/Cas9 is the most advanced and can be employed to correct point mutations, thereby eliminating the nonsense mutation at the genomic level [120]. Gene transfection strategies involve the introduction of a wild‐type copy of the mutated gene into cells, often in mRNA form. This approach restores gene expression and provides an alternative source of the functional protein [121]. Post‐transcriptional gene recovery relies on suppressing PTCs during translation. This is achieved through the incorporation of near‐cognate transfer RNA (tRNA), in correspondence with a PTC, allowing protein translation. Two main therapeutic strategies have been developed to support this process. One involves the use of anticodon‐engineered tRNA (ACE‐tRNA), a modified aminoacyl‐tRNA designed with a specific anticodon to recognize and suppress the PTC [122, 123]. The other strategy utilizes a class of compounds known as translational readthrough‐inducing drugs (TRIDs), which pharmacologically promote PTC readthrough [124]. A last alternative post‐transcriptional approach focuses on inhibiting nonsense‐mediated mRNA decay (NMD), increasing the cytoplasmic mRNA pool that can undergo basal‐level translational readthrough [125].

## 4 Conclusion

Nonsense mutations are severe genetic alterations and are responsible for some rare and ultra‐rare genetic disorders. These mutations lead to the premature termination of protein synthesis, often resulting in severe phenotypes due to the loss of essential cellular functions. They represent a significant challenge in the treatment of NRDs, and a deeper understanding of nonsense mutation molecular mechanisms and their impact on cellular pathways will be crucial in developing and improving therapeutic strategies, ultimately aiming to rescue protein expression and cellular function. Advancements in molecular biology and genetic therapies have paved the way for promising therapeutic approaches to counteract the detrimental effects of nonsense mutations. Innovative strategies, such as pharmacologically induced translational readthrough, gene editing, mRNA‐based therapy, and targeted pharmacological interventions, hold great potential for restoring functional protein expression and alleviating disease severity. However, further research is needed to optimize the current proposed treatments and to find new strategies.

## Conflicts of Interest

The authors declare no conflicts of interest.
